# Ninth Annual Meeting of the American Society of Dental Surgeons

**Published:** 1848-10

**Authors:** 


### Miscellaneous Notices.

### NINTH ANNUAL MEETING OF THE AMERICAN SOCIETY.

The ninth annual meeting of the American Society of Dental Surgeons was held, Aug., 1848, at Saratoga Springs.

The meeting convened at the Union Hall, on Tuesday, Aug. 1st, at 12, M., the President in the chair.

The Recording Secretary read the minutes of the last meeting, which were accepted and approved.

The roll being now called, the following gentlemen answered to their names, viz. Drs. E. Parmly, C. A. Harris, J. A. Cleveland, J. Parmly, J. H. Foster, Robert Nelson, Wm. H. Dwinelle, J. B. Rich, E. J. Dunning, C. O. Cone, Asa Hill, A. Westcott.

Letters were read from several gentlemen, giving special reasons why they could not be present at the meeting.

The Executive Committee were now requested to prepare the order of business, &c., and the Society adjourned to 3½ o'clock, P. M.

*3½ o'clock, P. M.*

The Society convened agreeably to adjournment.

The Executive Committee reported the following order for this afternoon, viz.

1. To hear the report of the committee on *practical dentistry*.
2. General discussion of practical subjects.
3. Election of officers.

The committee on practical dentistry was now called upon for their report. Dr. C. O. Cone, the chairman of the committee, stated that the committee had been unable to convene to examine the report, and he therefore hoped that time would be allowed them to do so before it was read. On motion, the reading of the report was therefore postponed until the next morning.

The Society then took up the next business in order, viz. *the discussion of practical subjects*.

The first subject introduced was the *filing or otherwise separating teeth, as a preparatory step to plugging*.

As this discussion was taken at length by a *short-hand* reporter, who had been employed by the Society for the purpose, the Recording Secretary deemed it unnecessary to keep any minutes of this part of the proceedings, farther than the order adopted. This discussion was continued till 7, P. M., at which time the Society adjourned to 8, P. M.

*Tuesday*, 8, *P. M.*

The Society convened agreeably to adjournment.

Before the Society proceeded to the election of officers, it was moved that the resignations of several members be considered.

1. Was that of *Lewis Roper*, which was unanimously accepted, his dues having been paid to date.

2. Was that of *C. C. Allen.* It was moved and seconded that his resignation be accepted, on his complying with the requirements of the constitution, in paying his dues. Carried.

3. That of *C. S. Rowell* was accepted on the same conditions.

4. That of *John W. Hawes* was also accepted on the same conditions.

It was moved and seconded that Dr. L. Roper's letter of resignation be published with the proceedings of the meeting. Carried.

*To the President of the American Society of Dental Surgeons:*

Dear Sir :—I beg you to communicate to the members of the American Society of Dental Surgeons, that circumstances oblige me to resign my office as Vice-president of the Association, and to withdraw myself altogether from membership in their useful and respectable body.

In offering my resignation, permit me, Mr. President, to tender to my former colleagues my grateful acknowledgments for the high honor they have conferred upon me, and to express the deep regret I feel in estranging myself from a professional association, for the members of which I entertain the greatest respect and consideration.

Very respectfully, your servant,

LEWIS ROPER.

Philadelphia, *Dec.* 1*st*, 1847.

The Society now proceeded to the election of officers, and with the following result, viz.

| | |
|---|---|
| E. PARMLY, | President. |
| J. A. CLEVELAND, | 1st Vice-President. |
| E. TOWNSEND, | 2d " " |
| ALEX. NELSON, | 3d " " |
| A. WESTCOTT, | Recording Secretary. |
| J. B. RICH, | Corresponding " |
| C. O. CONE, | Treasurer. |
| J. H. FOSTER, | Librarian. |
| C. A. HARRIS, | 1st Editor. |
| A. WESTCOTT, | 2d " |
| WM. H. DWINELLE, | 3d " |

*Publishing Committee.*—The Editors of the Journal.

*Executive and Examining Committee.*—J. H. Foster, J. B. Rich, E. J. Dunning, E. Parmly, J. M'Ilhany, C. A. Harris, H. N. Fenn.

*Opening Address.*—S. P. Hullihen.

Adjourned to 9, A. M., to-morrow.

*Wednesday*, 9, *A. M.*

C. O. Cone, Chairman of the Committee on Practical Dentistry, presented his report, the other members of the Committee having failed to assist him in the discharge of the duty assigned the Committee.

Dr. Cone was requested to read it, which occupied several hours. The Society now adjourned to 4, P. M.

*Four o'clock, P. M.*

Dr. Cone offered his resignation as a member of the Committee on Practical Dentistry. Accepted. Dr. J. H. Foster also offered his resignation as a member of the same Committee. Accepted.

On motion of J. Parmly, the Committee on Practical Dentistry was reduced to three members, which are, J. B. Rich, E. Maynard, and Alex. Nelson.

On motion of Dr. Harris, a Committee was appointed on Dental Literature and Education. This Committee was appointed by the Chair, and is as follows: S. P. Hullihen, C. A. Harris and Robert Arthur.

On motion of J. B. Rich, it was

*Resolved,* That three members of this Society be appointed by the Chair, to attend the National Medical Society,

*Delegates*—Drs. E. Maynard, A. Westcott, J. H. Foster.

The Society now proceeded with the discussion of practical subjects. Dr. E. Parmly, being requested to do so, gave the origin of the application of wedges for separating teeth, preparatory to plugging.

*Thursday, 9, A. M.*

The Society met agreeably to adjournment.

The first business which was introduced was the disposition of several cases laid over last year.

The first called up was that of E. M. Bissel.

Dr. Bissel was not present, but his name was answered to by Dr. John A. Cleveland, who stated that Dr. Bissel *did* return the protest, and also his annual dues, but the letter miscarried and was sent to Washington, and finally returned; that he was fully prepared to vouch for him in every particular; his case was therefore laid over another year.

G. W. Parmly's case was also laid over another year, he being still out of the country.

The case of Dr. James Taylor was next called up, and a response made by C. A. Harris. Dr. H. stated that Dr. Taylor had gone upon the supposition that his signature to the original protest in 1845, which was full and strong, was all that was required, and that he had hence neglected to sign or return the one sent him; that he had good reason to believe Dr. T. to be right, both in theory and practice, upon this subject. His case was therefore directed to be laid over another year, with directions to the recording secretary to address Dr. Taylor upon the subject.

Dr. Thos. P. Cleveland tendered his resignation, on the ground of having retired from business, in consequence of a partial loss of sight. Accepted.

Dr. C. A. Harris then proposed him as an honorary member, and he was duly elected as such.

Dr. C. O. Cone offered his resignation as treasurer, which was accepted, and E. J. Dunning appointed in his stead, with instructions to employ a *collector* in all cases where it was inconvenient for him to attend to collections in person.

Some discussion was here had in regard to a resolution adopted at last meeting, relative to *patents* and *secrets* being held by members of the Society.

It was contended by several members, that while they did not wish nor intend to advocate either secrets or patent rights, as connected with direct and actual practice of dental surgery, they deemed the resolutions of last year quite too general in their scope, and several amendments were offered. It was finally decided to rescind them altogether, and prepare a new resolution, which would more definitely cover the ground intended by the resolution.

The *tabular sheet* which had been prepared by Dr. Cone, was now exhibited and explained by him.

The Society requested Dr. Cone to have one hundred copies struck off for the benefit of the members, and each member was requested to record upon it his cases, and return it to Dr. Cone by the first day of May next.

Adjourned to 3½, P. M.

*Thursday,* 3½, *P. M.*

The corresponding secretary reported progress in regard to the duty assigned him of addressing the honorary members upon the subject of amalgams.

The following members were appointed to deliver addresses at the next meeting: Drs. A. Westcott, J. H. Foster, C. A. Harris, W. H. Dwinelle and C. O. Cone.

The discussion of practice was here resumed, and continued till seven o'clock, at which time the Society adjourned to meet at half past 8, P. M.

*Thursday,* 8½, *P. M.*

The evening session was occupied till a late hour in discussing matters of practice. Dr. Asa Hill having expressed a desire to make some communication in regard to his gutta percha stopping, he was called upon to do so. But it appeared very soon that instead of wishing to enlighten his brethren in regard to the compound, he only wished to bring to notice its virtues. After Dr. H. had proceeded so far as to make his object fully apparent, he was asked whether he intended to give to the society or to the world, his receipt. To this he replied that he was *not at liberty at present* to do so, being equally concerned with another individual, whose interest he could not compromise. The question was then raised, whether it was proper for the Society, as such, to listen to his mere recommendations of his secret compound. This was soon decided in the negative, and the doctor deferred his remarks till after the Society should have adjourned.

After some discussion as to the most appropriate place for holding the annual meetings, or at least the next one, it was finally decided to adjourn to meet again at Saratoga Springs, on the first Tuesday of August, 1849.

